# Does remission of type 2 diabetes matter? A qualitative study of healthcare professionals' perspectives and views about supporting remission in primary care

**DOI:** 10.1111/dme.15515

**Published:** 2025-01-18

**Authors:** Mireille Captieux, Bruce Guthrie, Julia Lawton

**Affiliations:** ^1^ Usher Institute, Medical School University of Edinburgh Edinburgh UK; ^2^ University of St Andrews North Haugh St Andrews UK

**Keywords:** health care delivery, primary care, qualitative methods, type 2 diabetes

## Abstract

**Background:**

Trials conducted in highly selected populations have shown that type 2 diabetes (T2D) remission is possible, but the feasibility and acceptability of supporting remission in routine clinical practice remain uncertain.

**Aim:**

We explored primary care professionals' perceptions and understandings of T2D remission and their views about supporting remission within routine clinical care.

**Methods:**

Semi‐structured interviews were conducted with 14 GPs and nine nurses working in Scottish general practices. Data were analysed thematically.

**Results:**

Most participants considered remission to be a motivational tool but were unsure that it actually altered clinical management, due to patients still requiring follow‐up and their expectations that remission is often temporary because of the constant effort required to sustain remission in an obesogenic environment. These perceptions, together with participants' concerns about loss to follow‐up of patients who were likely to relapse and/or were still at high cardiovascular risk, appeared to underpin a reluctance to code remission in medical records. Most participants did not consider remission support to be a clinical priority. Moreover, they described being sensitive to the pitfalls of only encouraging some patients to pursue remission, because if resources were directed towards apparently more motivated, affluent individuals, there was a risk that this could widen health inequalities.

**Conclusion:**

For integration of remission support into mainstream T2D care to be successful, primary care professionals may need to be persuaded that remission matters more than encouraging well‐managed T2D. They would also benefit from clear guidance on follow‐up and optimal support for people in remission.

**What's new?**

- The DiRECT trial has shown that remission of type 2 diabetes is possible.
- However, supporting remission in everyday clinical practice is likely to present distinctive challenges and will require buy‐in from primary care professionals (PCPs). Despite this, limited research has explored their perspectives.
- We found that PCPs did not consider remission to be a clinical priority. PCPs also reported that remission did not change their approach to clinical management.
- PCPs may need to be persuaded that remission matters more than encouraging well‐managed type 2 diabetes. PCPs need clear guidance on follow‐up and optimal approaches to support people in remission.

## 1 INTRODUCTION

Type 2 diabetes (T2D) is a serious and common health condition that often leads to micro‐and macrovascular complications (e.g., retinopathy, ischaemic heart disease) due to suboptimal glucose management.^1^ T2D places considerable burdens on both the individuals who have it and wider society. By 2035, the cost of managing T2D in the United Kingdom (UK) is estimated to reach £35.6 billion.^2^

Research studies use a wide range of definitions for remission, and there is no universal consensus, but remission can be defined as a return to normal glycaemic levels (HbA1c <48 mmol/mol) for 3 months without the use of glucose‐lowering treatment (GLT).^3^ Maintaining normal glycaemic management without using drugs or risking drug side effects is an attractive and cost‐effective prospect for both individuals and wider society.^4^ Trials and observational studies using total meal replacement have shown that remission of T2D is a practical target in a primary care setting. However, participating volunteer primary care professionals (PCPs) did receive considerable training and support from study research dieticians.^5^, ^6^, ^7^, ^8^ Supporting remission in everyday general practice, where the vast majority of people with T2D receive their care, is likely to present new and additional challenges and will require buy‐in from those tasked with this responsibility.^9^ However, to date, limited work has explored PCPs' perspectives and views about supporting T2D remission in routine care as opposed to in a research setting, such as a trial of the DiRECT intervention,^10^, ^11^ or a pilot of low‐calorie diet program.^12^ An exception is Alsaeed et al., who undertook focus groups with Kuwait‐based dieticians.^13^ However, only some of their participants were based in primary care, and study insights may have limited relevance to the United Kingdom (UK) due to differences in culture and healthcare delivery in the two settings. Hence, further work exploring PCPs' perspectives and views about supporting remission in routine clinical care is needed.

To address this gap, we undertook an interview study with PCPs based in Scotland, UK. The aim of this interview study was to understand and explore PCPs' perceptions and understandings of remission and their views about supporting remission within routine clinical care. Our objective was to consider the implications of PCPs' perceptions and understandings for integrating remission support into mainstream T2D care.

## 2 METHODS

### 2.1 Overview

As little was known about the area under investigation, we employed an exploratory, inductive design, which allowed findings and themes to emerge from the data rather than testing pre‐determined hypotheses.^14^ To achieve this, we used semi‐structured interviews informed by a topic guide that comprised a series of open‐ended questions. This design allowed our interview discussions to explore areas relevant to addressing the study aims while enabling us to tailor particular questions to particular participants (e.g., according to their job role) and offering the flexibility needed for participants to raise and discuss issues they considered salient, including those unanticipated when the study began. Consistent with an inductive, exploratory approach, we undertook data collection and analysis concurrently and refined/revised our topic guides as we went along to allow (unanticipated) findings identified in early interviews to inform areas explored in later ones.^15^

Ethical approval for this study was granted by the Usher Research Ethics Group (Ref: 2011, date 18/12/20).

### 2.2 Recruitment and sample

We recruited PCPs from general practices across Scotland. Our sampling strategy aimed to attain a breadth of experience and views, including outlier perspectives. This was reflected in our use of broad inclusion criteria, namely, any GP or practice nurse (PN) who clinically supports people with T2D in routine care. As we recognised that contextual factors might influence how PCPs thought about and used the concept of remission,^16^, ^17^ we also sought to attain a diverse sample in terms of practice location (rural or urban), practice population (deprived or affluent), and participant experience and job role (e.g., PNs; sessional, salaried, GP partners; GPs or PNs with a special interest in diabetes).

To help achieve a diverse sample of participants from across Scotland, we used three recruitment strategies: (1) We circulated recruitment packs, including opt‐in forms, by email to GP and PN networks across Scotland. (2) We used publicly available contact details (e.g., practice email address or phone number) to contact practice managers, cluster quality leads, or primary care diabetes leads; these individuals were given recruitment packs to pass on to GP and PN colleagues. (3) We also used snowballing approaches wherein we asked existing study participants/colleagues to pass on recruitment packs to individuals we identified as being needed to meet our sampling aspirations (e.g., those working in types of practices or job roles currently under‐represented in our sample). Throughout the study, we kept a recruitment log, which we reviewed regularly to help ensure our sampling goals were attained. We continued to recruit until our ongoing reviews of our interviews (undertaken by all team members) confirmed that data of sufficient depth and breadth had been collected to address the study aims.

### 2.3 Data collection

Interview topic guides were developed in light of literature reviews, prior work involving defining remission,^3^ and examining the prevalence of remission in Scotland^18^ and input from primary care colleagues. Topic guides were revised in light of emerging findings, in line with the study's inductive approach (see Table 1 for an overview of the main areas explored relevant to the reporting in this article). Interviews were conducted by MC, an academic GP with qualitative training, with mentorship and support from JL (an experienced non‐clinical qualitative researcher), who jointly reviewed interviews with MC and was involved in decisions about topic guide revisions/refinements. All interviews were undertaken remotely using MS Teams between November 2021 and March 2022. Interviews lasted 40–90 min, were audio recorded, and transcribed in full for in‐depth analysis.

**TABLE 1:** Topic guide.

*Introduction*
*General–to contextualise findings*
What size is your primary care centre, e.g., how many people with type 2 diabetes does it support, how many staff are there, and what are their roles?
Can you describe your practice population/catchment area (prompts: do you service a predominantly affluent or poor population, and do a lot of people from ethnic minority backgrounds attend the practice?)
Is this practice different from other practices, in what respect?
Could you tell me about your role(s) in the practice and professional background? How many years have you been practising? (Explore; do they have a special interest in diabetes?If so, what led them/motivated them to work with people with diabetes? If they do not specifically have a diabetes role, then what are their thoughts on managing people with diabetes? Does anyone else manage people with diabetes?
Can you describe what it is currently like to work in primary care? (Prompt: What factors drive you to continue supporting people with diabetes in this job? Has your job changed since you started working in primary care? In what ways? Has the way in which you care for and manage people with T2D within your practice changed over time?)
*Experiences of remission of diabetes*
What is the first thing that comes into your mind when you hear “remission of diabetes”? (Prompts: When did you first hear about remission? What were your initial thoughts? Have your thoughts changed since then? Why? (where did your information/knowledge come from?)
Have you diagnosed and coded remission? (Explore which code is used and what precise circumstances would need to be fulfilled for an individual to be diagnosed/coded with remission. [Encourage the interviewee to describe their understanding of what remission actually is (clinical criteria); if descriptive, how/where would they get numerical thresholds?] How confident do you feel about diagnosing and discussing remission with people with T2D? (What things are unclear and why?)
Do you feel that you share a common understanding with other people in your practice about what remission is and whether it should be supported by primary care? Do you agree on who should take responsibility for supporting remission? (What factors influence your similar/different approaches to remission?)
What benefits do you think people with type 2 diabetes and their carers/family can get from remission?
Do you think some people are better candidates for remission support? Which kinds of people, and why?
Based on your experiences to date, do you have any concerns about how the concept of remission might affect the people you care for, you, or the clinic? What are these concerns? (Prompt: Do you think it is realistic?)
*The implementation of diabetes remission in primary care*
What is the future of diabetes remission? (prompt: Do you see remission integrating into existing diabetes management pathways? What opportunities or challenges does remission present for people with T2D, you personally, and other healthcare professionals?)
Do you think the concept of remission has already led to any changes in your practice population, you, or the practice?
*Closing the interview*
Is there anything else you want to add that you haven't had the opportunity to say? Thank the participant for their time.

### 2.4 Analysis

To help achieve rigour and minimise the risk of researcher bias, a clinical (MC) and non‐clinical qualitative researcher (JL) were involved in data analysis. MC and JL analysed all interviews thematically using the method of constant comparison.^15^ Interviews were read through repeatedly (data immersion) and cross‐compared to identify cross‐cutting inductive and deductively generated themes. Attention was also paid to potential differences according to, e.g., participants' clinical role and practice environment. The analysis was led by MC with JL checking and validating themes and encouraging reflexive discussions, such as on the potential ways in which MC's own identity as an academic GP might have influenced her interpretation of the data. As there was strong agreement between the two researchers as to what the overarching themes were, there was no requirement for third‐party arbitration. Data were then coded according to these overarching themes. Coded datasets were subject to further analyses to enable identification of subthemes and more granular data interpretations. NVivo 20 (QSR International, Doncaster, Australia) software was used to facilitate data coding and retrieval.

## 3 FINDINGS

The final sample comprised 23 participants, including nine PNs and 14 GPs working in a variety of practices and roles (Table 2). Below, we begin by reporting PCPs' definitions of remission before going on to consider PCPs' understandings of remission and how it relates to type 2 diabetes. We conclude by considering the ways in which PCPs' understandings of remission affected their clinical practice. To safeguard anonymity, pseudonyms indicating participants' job roles (e.g., GP, PN) are used throughout. As responses did usually differ according to participants' gender, job role, or practice location, this information is not included in our reporting below unless otherwise indicated.

**TABLE 2:** Characteristics of participating primary care professionals.

	Practice nurses *N* = 9 (all women)	General practitioners *N* = 14 (6 women)	All participants *N* = 23 (15 women)
Location			
Rural and remote	1	2	3
Accessible rural	1	1	2
Accessible small towns	0	2	2
Other urban area	2	1	3
Large urban area	5	8	13
Practice neighbourhood			
Deep end	0	2	2
Deprived	2	1	3
Mixed	5	9	14
Affluent	2	2	4
Job title			
Practice nurse	8	0	8
Lead diabetes nurse	1	0	1
Sessional GP	0	1	1
Salaried GP	0	3	3
GP partner	0	4	4
GP with special interest in diabetes	0	5	5
Retired GP	0	1	1
Involvement with diabetes review			
Involved in annual review	9	5	14
Not involved with annual review	0	9	9

### 3.1 Formal ways of defining remission of T2D

All PCPs broadly defined remission as normal glycaemic parameters in the absence of GLT. However, most emphasised that more granular definitions were not needed or relevant to their everyday clinical practice because a diagnosis of remission did not change their approach to clinical management:

I don't think the definition would necessarily change management, and that's more the crux of it for me… obviously the longer the better from the patient's perspective. (GP4)

Nonetheless, most did suggest that remission was a useful concept or “tool in our toolbox” (PN22) to use to motivate some individuals to make or sustain lifestyle changes by helping to disrupt a default trajectory of passive acceptance and/or worsening health and polypharmacy:

You're going to sit on a trajectory of increasingly taking tablets, increasingly adding more complications, finally ending up on insulin, and then… inevitably getting all the complications. I feel that actually remission is a real opportunity for people to actually engage with self‐management… it is actually not just an untreatable chronic disease. (GP1)

Whilst remission was considered a potentially positive and empowering concept, many PCPs (irrespective of a special interest in remission) described being less concerned about whether people actually achieved remission criteria. Instead, PCPs highlighted the physical and mental health benefits that could result from individuals being motivated to improve their glycaemic management without GLT:

You're not using medication to drive the blood sugar down. You're using a much more holistic health intervention. Because if you lose weight and you get fit, that's good for your cardiovascular system; it's good for your mental health. (GP18)

### 3.2 Diabetes is permanent; remission is temporary

Most PCPs did not view remission as a cure and suggested that a diabetes diagnosis should be considered permanent, irrespective of remission status:

I think I'm a bit clearer now in the fact that it's not your diabetes going away, and it will never go; you'll always have diabetes, but you can put it into remission. (PN20)

Indeed, many described how T2D (particularly a prolonged duration of active diabetes) conferred a risk of long‐term complications that could not be totally mitigated by remission:

From a cardiovascular risk perspective, you've still got the risk rate you've had of having a lifetime of diabetes; that's not gone away. So, I'm not going to stop your statin, and I'm not going to stop controlling your blood pressure. You might not be on any oral hypoglycemics anymore, but… (GP6)

Furthermore, almost all PCPs alluded to an expectation that most individuals would eventually relapse back into T2D because maintaining remission was challenging and required ongoing effort:

It's only in remission as long as you keep the weight off. So that, if you go back to your previous weight, then you're at risk of developing it again… and, my experience over the years is that people find it very difficult to change their lifestyle and keep it changed. (GP18).

To justify these claims, PCPs often provided detailed descriptions of aspects of the social and physical environment–what some termed the obesogenic environment–that made it difficult for people to initiate and sustain remission. Such PCPs also described feeling powerless to change the environment and social norms because these were largely out of their own and people with T2D's control. Indeed, as one PCP suggested, *“it seems obvious that it's a public health narrative, not a clinical narrative”* (GP4), whereas another reflected on the very real difficulties of initiating and sustaining lifestyle change in individuals living in adverse and impoverished circumstances:

So you could say, “Here's the gym membership for you; go and exercise”. But that's hard work, and people generally are working very hard. You know, either both adults will be working in a household and find it difficult to invest in their lifestyle. Habits of food and eating have been developed over 20, 30 years; the shops sell what they sell. (GP3)

### 3.3 A reluctance to code remission of T2D

UK GP electronic medical records are coded using Read codes (a bespoke UK coding system for primary care). At diagnosis, entry of any of a standard set of diabetes mellitus Read codes into the GP record means that the person is automatically added to the national diabetes registry (SCI Diabetes) and is recalled for annual retinopathy screening (coordinated and performed outwith primary care). Practices also use these codes to run internal registers and systems to recall people for structured review. However, whilst being encouraged to code remission in medical records, most PCPs expressed a strong reluctance to do so due to their perceptions of it being a transitory state that still conferred risk of long‐term complications. In contrast, many GPs described a strong clinical obligation to label electronic medical records with a T2D Read code at diagnosis and to preserve this code to help ensure the individuals concerned continued to be regularly monitored:

You can bring someone down into healthy HbA1c, but as GPs we keep them coded so that they get their annual eye follow‐up, and we will probably still bring them in annually to check the HbA1c and do their risks, their profile, their cardiovascular risk, and things like that. So even though they're in remission, they still get treated like a patient. (GP3)

Such PCPs expressed concerns that coding diabetes in remission might mean individuals would be “*lost to follow‐up*” (GP6), with others noting that remission was likely to be too transient to warrant the “*thought, time, and effort into worrying about changing what we do with them*.” (GP3)

### 3.4 People currently in remission are not a clinical priority

PCPs further described how they did not tend to prioritise people in remission for any specific support to maintain remission. This is partly due to their perceptions that people in remission were highly motivated and required minimal support to initiate their remission, and who were “*the ones I don't worry about*.” (PN23). Furthermore, the vast majority reflected on the multiple challenges and competing priorities they faced in their everyday clinical practice, which, as some further observed, had been exacerbated by the SARS‐CoV‐2 pandemic. Such PCPs described being overwhelmed by the deterioration in glycaemic management in previously stable people with T2D, together with increased acute clinical demand and frequent staff shortages and illness, all of which, they suggested, had resulted in them having limited time to meaningfully support remission within their practice:

In terms of priorities, of course, my patients who were in remission before the pandemic have been way down on the priority list, haven't they, to chase up and to do their reviews? (GP21)

### 3.5 Identifying candidates for formal remission support

As well as highlighting time and staffing constraints, PCPs discussed how motivating and encouraging remission in certain individuals could have negative consequences, such as inadvertently stigmatising them or damaging the therapeutic relationship by inappropriately imposing remission on them and setting unrealistic goals.

I also feel remission is… it's a double‐edged sword. So, if you achieve it, fantastic, but if you aim for it and totally fail to get it, it can be very demotivating. (GP19)

Given these constraints and concerns, many PCPs described focusing on those individuals who they felt would be most receptive to, and have the most to gain from, intensive lifestyle support, rather than on increasing the absolute numbers of people in remission. In this context, PCPs commonly mentioned age; specifically, they described how putting diabetes into remission had more potential health gains for younger people:

The longer you live with an uncontrolled HbA1c or uncontrolled blood sugar in general, the more likely you are to have complications. So my target going forward is really young people. So we're seeing more young people diagnosed with type 2 diabetes at younger ages as a result of obesity, usually; obesity feeds in there as well. But that's the group of people that really worries me. (GP17)

Some PCPs suggested that remission would have less beneficial effects on older or frailer people because older people recently diagnosed with diabetes might never be affected by the long‐term complications. Others observed that older people who had had diabetes for a “*lifetime*” would have already accumulated a significant cardiovascular risk burden upon which remission would have little effect. Some PCPs further suggested that older people might have a decreased capacity or motivation for lifestyle change due to comorbidity (e.g., severe osteoarthritis) or a resistance to change:

… overweight with bad osteoarthritis… half of them are in their 80s. You know, you're not going to go out and do exercise to lose weight; that's just not achievable, so there's no point in trying to push this. (GP3)

Returning to their concerns about the obesogenic environment, many PCPs suggested that living during a time of austerity or in conditions of deprivation was not conducive to the behaviour change required for people to put their diabetes into remission. This issue was voiced most explicitly and sensitively by PCPs who worked in areas of high deprivation:

‘Just give me the pills, doctor, what on earth are you talking about?, remission, I've no idea what you mean. There's no way I can make any changes in my lifestyle, no.’ There is people who don't have the capacity to make those choices because they're using the food bank already, so they don't have that ownership over their dietary intake necessarily; they can't make a choice to have a healthy diet. (GP12)

However, many PCPs also highlighted that it was difficult to predict who would successfully achieve remission and that any patient had the capacity to surprise, whatever their characteristics and circumstances. Hence, PCPs, especially those working in deprived areas, were very clear that if remission support was only offered to those with the highest chance of success, then those with the most need would end up *“at the bottom of the heap*”:

I wouldn't do that because of my work with drug misusers, because I'm aware that you should never make judgements about people based… or it's a tricky judgement to bet on whether somebody's going to succeed or not and to alter your treatment accordingly. So, I think that I would try to encourage, but there's also got to be that thing about seeking to understand; it's about seeking to understand what's really happening with them. (GP15)

## 4 DISCUSSION

In this article, we have identified multiple factors that appeared to influence how PCPs perceived T2D remission in their everyday clinical practice. As we have shown, PCPs did not see particular value in having a precise definition of remission, and, furthermore, many did not make a clear distinction between remission and T2D, with some referring to remission as ‘well‐managed’ T2D. We also found that PCPs often perceived remission as being a temporary intermission from T2D, rather than a permanent state. Arguably, such observations reflect the fundamental challenges of defining a disease state by categorising a continuous laboratory measurement (HbA1c),^3^ alongside the difficulties, as others have also noted,^7^, ^8^, ^9^ of maintaining remission in the long(er)‐term.

PCPs also reported that adding the Read code for remission to medical records did not alter care, because both groups had the same management and follow‐up plan, and some PCPs also described being unsure whether remission coding would affect recall systems in problematic ways. PCPs appeared to value remission as a motivational tool or concept that could be used to shift the focus of diabetes management from progressive polypharmacy to lifestyle change, with the potential to contribute to patient activation and self‐management irrespective of whether remission was actually achieved.^19^ However, they also voiced concerns about the potential to reinforce existing health inequalities if resources are directed at affluent and seemingly more motivated individuals who might have more capacity to respond to, but not necessarily the most to gain from, remission support.^20^

Similar to our own findings, Wylie et al. found that ambiguity in how a diagnosis of pre‐diabetes affected clinical management made GPs uncertain about the value of such a diagnosis.^21^ Alsaeed et al.*'s* qualitative study in Kuwait likewise found that while dieticians considered remission to be motivational, most tended to focus on achieving weight loss or people with T2D's own goals rather than attaining remission per se.^13^ In relation to coding, others have suggested that poor or variable remission coding in primary care might be due to PCPs lacking IT skills or disliking the administrative burden that coding entails.^22^, ^23^ In contrast, our findings suggest that the key consideration for PCPs is to fulfil a duty of care and avoid unintended harm, such as loss to follow‐up. Indeed, PCPs described being very careful to add and preserve the T2D Read code because they believed it was still important to continue screening for diabetes complications (particularly eye screening) and cardiovascular risk management.

PCPs' accounts are also consistent with the hypothesis that living in the modern obesogenic environment facilitates development and maintenance of obesity,^24^ and resonate with previous qualitative work exploring GPs' and PNs' views about weight management.^25^, ^26^, ^27^ This work found that, while PCPs understood the importance of tackling obesity, they were reluctant to get directly involved due to a perceived lack of success and the potential to set individuals up for failure,^25^ their perceptions of people's inability to change,^27^ and their concerns that obesity is a societal rather than a medical problem.^26^ Additionally, PCPs' suggestion that remission can be very challenging to maintain has been echoed in studies involving DiRECT trial participants, who similarly highlighted the effortful nature of remission maintenance and the need for ongoing encouragement and behavioural support from healthcare professionals.^11^, ^28^, ^29^ Importantly, these individuals also attributed successful attainment and maintenance of remission to the sense of accountability to the wider research team that resulted from taking part in a clinical trial.^28^

PCPs also highlighted the limited clinical benefit that they felt could result from supporting remission in certain groups of individuals living with T2D, such as older people who are usually excluded from clinical trials like DiRECT (where participant mean age was ~54 years).^5^ This perspective is consistent with literature that suggests that, while approximately 8 years of tight glycaemic management is necessary to improve vascular outcomes,^30^ many older, frail people with T2D have a life expectancy of less than 8 years, meaning that, potentially, remission is less relevant for them.^31^

One of the questions that emerged during this research is whether remission of T2D matters to PCPs. Our study suggests that, while PCPs were not fundamentally opposed to the idea of remission (indeed, some found it to be motivational for some people under their care), they did not usually consider remission support to be a clinical priority, especially given the increasing numbers of people with T2D and resourcing constraints. PCPs offered good reasons for why they did not usually code remission. Arguably, providing a consistent, easily accessible definition of remission along with unambiguous guidance on coding remission is a necessary first step to improving coding, but our findings also suggest that this is unlikely to be sufficient to change practice unless PCPs are persuaded that coding will support better care. Any large‐scale implementation program aiming to integrate remission into primary care diabetes management should therefore ensure that PCPs understand why they should be supporting patients to achieve remission rather than well‐managed T2D.

There is also a need for guidance about appropriate, structured follow‐up for people in remission, including how to support long‐term remission maintenance. This study showed that PCPs used remission as both an outcome target and a broader motivational tool. However, PCPs also identified multiple areas that they found confusing and problematic, including the place of remission in older and frailer people and guidance on when and how to stop GLT and preventive medication such as statins. It would therefore be helpful to create resources for PCPs that not only show the referral criteria for formal total meal replacement programs (similar to the intervention used in the DiRECT trial) but also alternatives for people who do not meet criteria or who do not wish to use total meal replacement and practical guidance on medication management.

Until then, it is important to recognise that coding is likely to remain variable, and, therefore, evaluations of weight management programs or the reporting of population‐level data should not rely on primary care coding of remission to identify people in remission. Instead, measurement using routine data should apply the underlying formal remission criteria (normoglycaemia for a minimum period of 3 months without glucose‐lowering therapy).^3^ Additionally, as our findings highlight, working with individuals to support remission will likely be of limited value if not accompanied by public health interventions to make the wider environment less obesogenic.^32^

This is the first UK‐based qualitative study reporting PCP's perspectives and experiences of supporting T2D remission in routine clinical practice. While the study successfully captured the perspectives of PCPs from a diversity of practices, data collection took place during the early phases of the SARS‐CoV‐2 pandemic, which may have influenced PCPs' perceptions, for example, about their ability to meaningfully support remission in primary care settings. However, given the ongoing crisis in primary care and difficulties recruiting and retaining GPs and other practice staff in the UK,^9^ it is likely that participating PCPs' concerns about supporting T2D remission remain highly pertinent. Additionally, as this study was based in the UK, some of the findings may not be generalisable to other countries where systems of health/diabetes care delivery may be different. This includes countries where healthcare is largely privatised as opposed to free at the point of delivery or where type 2 diabetes care is more specialist‐based.

Our findings indicate that there is a need for further research to define effective care pathways to maintain T2D remission in people who achieve it, including recall intervals and the intensity of dietary and weight management support. Research to identify the training and resources needed to run such care outside of clinical trials is also required. Additionally, several of the PCPs in this study pointed out that many of the people with T2D who they support are multimorbid, old, frail and/or living with the effects of deprivation. Hence, further research is needed to explore the benefits and logistics of initiating and supporting remission/weight loss in groups, such as those who have been largely excluded from trials of interventions to achieve remission. It also needs to be considered that this study focused on the perspectives of PCPs because primary care is the setting where most T2D care happens, but future research also needs to understand the views of other healthcare professionals involved in diabetes care, such as those based in secondary care. It is also crucial to explore the perspectives and experiences of people who are wanting or receiving remission support in primary care settings, as existing research, while extremely important, has only consulted those receiving remission support in the context of a clinical trial^28^, ^29^, ^33^ and who may, as a consequence, be particularly motivated and/or subjected to an inequitable referral process due to recruiter selection bias.^12^, ^33^ Finally, it should be noted that in Scotland, where this study was conducted, there are lower numbers of people from Black and minority ethnic backgrounds as compared to other parts of the UK. Hence, future work could consider the perspectives and experiences of PCPs supporting remission in underserved communities, where, as others have observed, cultural and language considerations may also influence whether healthcare professionals encourage and put individuals forward for remission support.^12^, ^34^

### 4.1 Conclusion

In summary, we have explored PCPs' perspectives on supporting remission in routine primary care. PCPs were not fully convinced about the importance of supporting remission for several reasons. Any large‐scale implementation of remission would need to address their reservations and concerns to help ensure PCPs are optimally engaged in supporting and sustaining remission in people with T2D.

## AUTHOR CONTRIBUTIONS

Mireille Captieux conceived and designed the interview study with input from Julia Lawton and Bruce Guthrie. Mireille Captieux collected the data, which was then analysed by Mireille Captieux and Julia Lawton. Mireille Captieux conceived the concept for this article and draughted it with input from Julia Lawton and Bruce Guthrie. All authors reviewed, edited, and approved the final version of the manuscript.

## FUNDING INFORMATION

MC was funded by Chief Scientist Office CAF 18/12 (https://www.cso.scot.nhs.uk/personal‐awards‐initiative/clinical‐academic‐fellowships/). The funder had no role in the study design, data collection, analysis, decision to publish, or preparation of the manuscript.

## CONFLICT OF INTEREST STATEMENT

The authors have no conflicts of interest to declare.

## CONSENT TO PARTICIPATE AND FOR PUBLICATION

All research participants provided written informed consent, including for anonymised information to be published in this article.

## Data Availability

The datasets generated and analysed in the course of this study are not publicly available due to risks to individual privacy. However, they are available, via the corresponding author, on reasonable request.
